# Multi-isotope analysis of bone collagen of Late Pleistocene ungulates reveals niche partitioning and behavioural plasticity of reindeer during MIS 3

**DOI:** 10.1038/s41598-023-42199-7

**Published:** 2023-09-21

**Authors:** Kate Britton, Elodie-Laure Jimenez, Mael Le Corre, Sylvain Renou, William Rendu, Michael P. Richards, Jean-Jacques Hublin, Marie Soressi

**Affiliations:** 1 Department of Archaeology, University of Aberdeen, Aberdeen, AB24 3UF UK; 2 Department of Human Evolution, Max Planck Institute for Evolutionary Anthropology, 04103 Leipzig, Germany; 3 Royal Belgian Institute of Natural Sciences, 29 Vautier Street, 1000 Brussels, Belgium; 4 Hadès Archéologie, Agence Atlantique, 33100 Bordeaux, France; 5 PACEA, UMR 5199, CNRS, Université de Bordeaux, Ministère de la Culture et de la Communication (MCC), 33400 Pessac, France; 6 ZooSCAn (IRL 2013) CNRS - IAET SB RAS, Novosibirsk, Russia; 7 Institut Français des études sur l’Asie Centrale (IFEAC) - UAR 3140 (CNRS-MEAE), Bishkek, Kyrgyzstan; 8 Department of Archaeology, Simon Fraser University, 8888 University Drive, Burnaby, BC V5A 1S6 Canada; 9 Collège de France, 11, Place Marcelin Berthelot, 74005 Paris, France; 10 Faculty of Archaeology, Leiden University, 2333CC Leiden, The Netherlands

**Keywords:** Palaeoecology, Archaeology, Stable isotope analysis

## Abstract

Here we present stable carbon, nitrogen and sulfur isotope ratios of collagen extracted from *Rangifer*, *Equus* and *Bison* bone (*n* = 128) from different stratigraphic levels at the chronologically well-constrained Middle and Upper Palaeolithic site of Les Cottés, France. Samples were taken from five phases of site use (US08, US06, US04 [upper and lower], and US02; ~ 45.8–35.3 ka cal BP) to explore the dietary and spatial palaeoecology of these ungulate species during MIS 3, and the contemporary climate. Temporal trends in *δ*^15^N values of all species broadly align with other climatic indicators at the site and the lowest values in US04 correspond to the Heinrich 4 cooling event, reflecting changes in the composition of soil/plant nitrogen at this time. *Rangifer* collagen is ^13^C-enriched compared to the other species throughout, consistent with lichen consumption. However, this isotopic niche partitioning between *Rangifer* and *Equus*/*Bison* is most extensive during US04, indicating plasticity in reindeer feeding behaviour, and potentially overall increased lichen biomass during this cooler/more arid phase. *Rangifer δ*^34^S values are consistently lower than *Equus* and *Bison*, which could be indicative of their more extensive spatial ranges incorporating greater inland areas. *Equus* and *Bison* demonstrate a significant decrease in *δ*^34^S values through time, which may be linked to contemporary climatic decline.

## Introduction

Stratified archaeological deposits in karstic caves and rock shelters, which are often rich in animal skeletal remains due to the subsistence behaviours of early human groups, represent excellent archives of potential information about the palaeoecology of past megafaunal species. Such materials are often the focus of detailed study as zooarchaeologists seek to reconstruct the ways in which hominin groups used animal resources^1–3^ and are often radiocarbon dated, as archaeologists strive to place sites within chronological frameworks^4–6^. Other analytical approaches can be employed on the faunal remains themselves, such as isotope analysis^7–9^. Commonly undertaken at archaeological sites to provide a baseline for the interpretation of human dietary isotope data, these approaches can also reveal the dietary palaeoecology of extinct and ancestral taxa and the trophic relationships that existed in ancient ecosystems^10–12^. Due to differences in photosynthetic pathway, plant functional type, physiology, and other factors^13–16^, these approaches can also be effective in revealing (isotopic) niche and resource partitioning within herbivore communities. These approaches have revealed the feeding behaviours of extinct species such as giant deer (*Megaloceros giganteus*)^17^, mammoth (*Mammuthus primigenius*)^18^, and woolly rhino (*Coelodonta antiquitatis*)^19^ as well as resource partitioning amongst cervids, equids and bovids^20–22^. Diachronic isotopic studies have recently begun exploring plasticity or conservation of feeding behaviours in herbivore species through time during the Late Pleistocene in both individual species and in groups of species^23–25^. When considered alongside climate proxy data and/or in combination with other isotope approaches, such as strontium or sulfur isotope analysis that are used to reconstruct past landscape use and seasonal movements^9^, such studies can illuminate the adaptability and resilience of mid- and large-sized herbivores on a scale far beyond modern ecology.

However, the stable isotope values of ungulate bodily tissues are not only influenced by their dietary choices, but also by a range of broader environmental factors, including atmospheric CO_2_ concentration, soil pH, rainfall, aridity, temperature, distance to the ocean, extent of glaciation, and soil pedogenesis which serve to influence the isotope values of plants^14,16,26^ and therefore herbivore feeders in both modern and ancient ecosystems^9,10,27,28^. The Late Pleistocene in Europe was characterised by numerous large-scale climatic oscillations^29,30^, which in turn influenced the isotopic values of ungulates^8,24,25,31–38^. Due to the dual influences of both broader climatic suite and behavioural ecology on the stable isotope ratios of herbivore bone collagen, extricating environmental from behavioural signals can be complex. With dietary isotope data, this is especially important when considering ungulate taxa that can feed flexibly (e.g., intermediate feeders such as bovids or cervids) or species that can exploit food resources with characteristic isotope ratios that other species cannot. For example, due to their lack of vascular system inhibiting fractionation through diffusion, lichens exhibit elevated *δ*^13^C values relative to typical C_3_ plants^39–42^ and modern studies have demonstrated that their consumption induces ^13^C-enrichment in the skeletal collagen and soft tissues of animals that consume them^41,43,44^. The exploitation of such low protein forage, facilitated by a unique gastrointestinal microbiome^45,46^, also allows reindeer and caribou (*Rangifer tarandus* ssp.) to occupy environments in which most other ungulates are unable to flourish^47^. Furthermore, plastic feeding behaviours (i.e., mixed feeding, ability to thrive on lichens), or indeed more extensive ranging habits/migrations, may increase with changing environmental conditions, serving to exacerbate or attenuate any isotopic trend within any one species. Multi-species studies are required to determine the extent to which these niche feeding behaviours change through time, and with climatic change.

**Figure 1:**
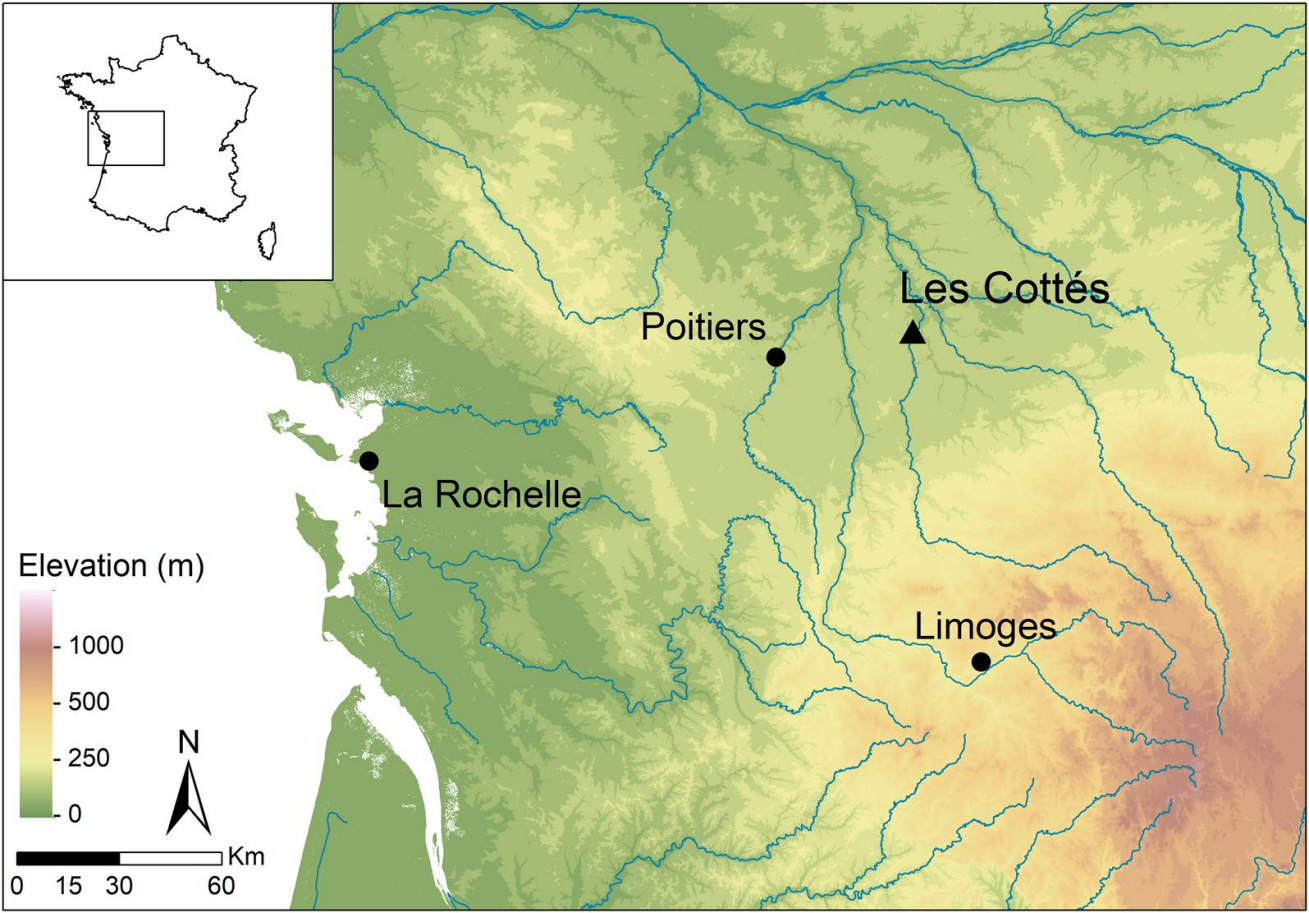
Topographical of the region around Les Cottés. Map created in ArcGIS 10.5.

**Figure 2:**
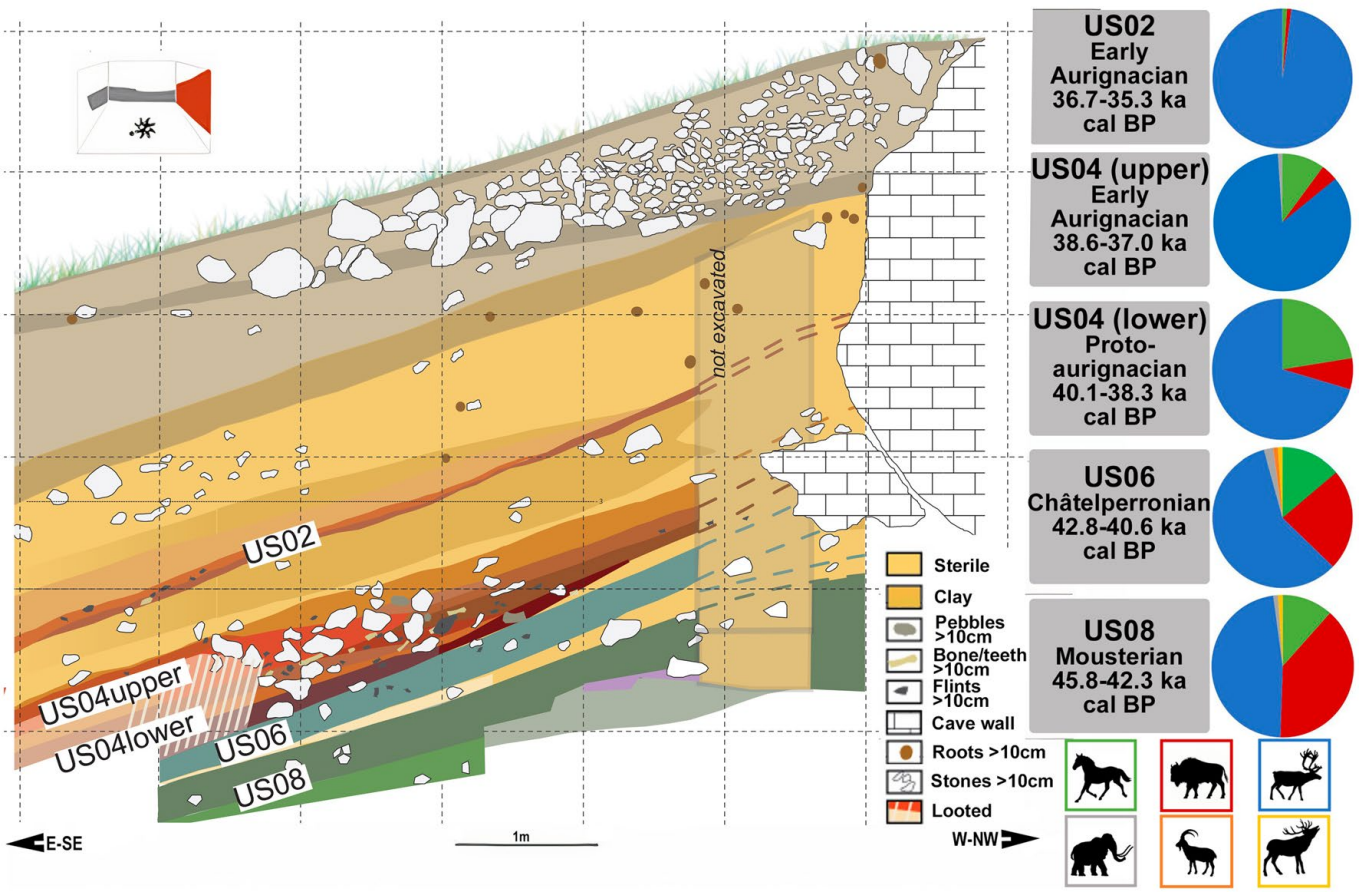
Stratigraphic units at Les Cottés, with details of dates and relative frequencies (%NISP) of medium and large size ungulates in the faunal assemblage, using previously published dates and faunal abundance data^2,4^.

Dating to between ~ 45 to ~ 35 ka BP, with good agreement between radiocarbon and OSL dating^4,48^, archaeological deposits at the rock shelter of Les Cottés, Saint-Pierre-de-Maillé, Vienne (Fig. 1), boast a large faunal assemblage, including reindeer (*Rangifer tarandus*), bovids (*Bison* sp.) and horses (*Equus* sp.) throughout the sequence. Of *Equus *specimens identified to species, *Equus ferus* predominates, with only two examples of *E. hydruntinus* described^2^. The site was utilised by both Neanderthals and Anatomically Modern Humans and, although there are differences in faunal species abundance through time (Fig. 2), the archaeological record of the site does not (currently) highlight any major differences in use, subsistence strategy or seasonality characterising occupations by these different hominin species. While large ungulates such as horses and bovids dominate the faunal assemblages in the oldest (Mousterian) levels (US08), reindeer increase from unit US04 (lower) onwards, corresponding with complete replacement of woodland/temperate species (red and roe deer, wild boar), and the rise in cold-adapted mountainous species (ibex, chamois) in units US04 (upper) and US02^2^ (Fig. 2). This pattern, of reindeer progressively replacing larger ungulates during this phase of MIS3 is seen at archaeological sites across many parts of western Europe, especially the Aquitaine basin and the Pyrenees^1,49^, and is consistent with the gradually cooling climate towards the Last Glacial Maximum^50^. The sequence at Les Cottés spans several climatic excursions during this period, including Heinrich event 4 (H4), a cooling event that occurred between 39 and 40 Ka, which was characterised by harsher and drier conditions in southern and western Europe^51^. At Les Cottés this is evidenced not only by the changes in ungulate species, but also by clay diagenesis throughout the sequence which attests to gradual climatic decline^52^. Species changes in micromammals at the site also point to climatic decline (Royer, unpublished data), which is consistent with evidence from some other sites, where the impact of the rapid climatic changes associated with the Heinrich and Dansgaard-Oeschger events are observed on a local, but not necessarily regional, level in southwestern France during MIS 1–3^53^.

The chronological well-constrained stratigraphy at Les Cottés is thus a rare example of a detailed continuous record spanning major climate fluctuations that occurred during late MIS3, offering the opportunity to study the palaeoecology of reindeer, horses and bovids over more than a 10,000-year period of the Late Pleistocene. In particular, the abundance of reindeer throughout the sequence permits the investigation of potential conservation or plasticity in dietary niche by this intermediate-feeding species, especially the consumption of lichens, in comparison to other grazing and intermediate feeding species (horses, and aurochs/bison). By restricting this investigation to a single site, other parameters, such as local ecological and geographical differences are also minimised.

The goals of this study are to explore and characterize plasticity in ungulate dietary ecology and range use over the period of site use and to analyse the intersections between these, climatic and environmental change. To achieve this, 132 bones from five phases of the site were targeted for collagen extraction and carbon, nitrogen and sulfur isotope analysis.

## Results

### Collagen preservation and sample integrity

Of the 132 bones sampled from the target levels, 128 provided sufficient collagen for analyses. The C:N of all samples and aliquots ranged from 2.9 to 3.6, with the majority of samples falling between 3.1 and 3.4, typical of terrestrial mammalian collagen, and suggesting well preserved collagen and isotope data likely reflecting in vivo isotopic composition^54–56^. However, data from a small number of bone samples (*n* = 7) were excluded from further discussion and analysis here due to low carbon content (< 30%) and low nitrogen content (< 11%) in data obtained from both laboratories^54^, leading to carbon and nitrogen data from 121 bone samples in total being included in data analyses. For sulfur, 126 specimens isotopic yielded data (having sufficient collagen for analysis) and all had collagen C:S values and N:S values corresponding with those expected for modern mammalian collagen (600 ± 300 and 200 ± 100 respectively^57^). However, from these analyses, 12 specimens were excluded due to % elemental data not meeting criteria^54^ at the analysing laboratory and 114 were used in the data analyses. All stable isotope measurements, along with all elemental data and sample information are provided in Supplementary Information (SI) Table S1 (including those excluded based on the above criteria) along with mean values for each sample (calculated from duplicate/triplicate/quadruplicate analyses). Given that we undertook multiple measurements of the same samples across two different laboratories, for the statistical analyses we utilised quality-checked data from the two analysing laboratories via mixed models (as opposed to models using mean values of multiple duplicates), using the analysing laboratory in the random effect term along with the sample ID. Estimates for each species were then compared two by two through pair-wise comparisons. The outputs of the mixed models are provided in Table S2-S4 and of the pair-wise comparisons by level in SI Table S5.

### Carbon (*δ*^13^C) and nitrogen (*δ*^15^N) isotope data

Mean carbon and nitrogen isotope ratios for the specimens analysed range from − 21.3 to − 18.6‰ and 3.0‰ to 10.4‰ respectively. Mean values by taxonomic groups are − 19.5‰ (± 0.4SD, *Rangifer*), − 20.4‰ (± 0.4, *Bison*), and − 20.6‰ (± 0.4, *Equus*) respectively for carbon, and 7.3‰ (± 1.2, *Rangifer*), 6.4‰ (± 1.4, *Bison*), and 5.8‰ (± 1.2, *Equus*) for nitrogen. Comparing all data across all levels, overall *δ*^15^N values in *Equus* are lower than *δ*^15^N values in *Bison* and (significantly so) compared to *Rangifer* (Table 1, Fig. S1b, with significance here and elsewhere referring to statistical significance specifically). *δ*^15^N values are also significantly higher in *Rangifer* than in *Bison* (Table 1). Considering all data from all levels together, *Rangifer* are also significantly higher in *δ*^13^C than the other two species (Table 1, Fig. S1a).

**Table 1:** Pairwise comparison of the estimates from the mixed linear models testing the differences in *δ*^13^C, *δ*^15^N and *δ*^34^S between species considering all levels together (all levels model: isotope value ~ species).

Isotope	Contrast	Estimate	SE	df	t-ratio	p-value
δ^13^C	*Bison—Equus*	0.25	0.108	125	2.32	0.06
	***Bison—Rangifer***	**− 0.83**	**0.093**	**125**	**− 8.92**	** < 0.001**
	***Equus—Rangifer***	**− 1.08**	**0.091**	**125**	**− 11.80**	** < 0.001**
δ^15^N	*Bison—Equus*	0.61	0.319	124	1.91	0.14
	***Bison—Rangifer***	**− 0.97**	**0.275**	**124**	**− 3.51**	**0.002**
	***Equus—Rangifer***	**− 1.58**	**0.272**	**124**	**− 5.79**	** < 0.001**
δ^34^S	*Bison—Equus*	0.81	0.640	117	1.27	0.42
	***Bison—Rangifer***	**2.29**	**0.558**	**117**	**4.11**	** < 0.001**
	***Equus—Rangifer***	**1.48**	**0.551**	**117**	**2.68**	**0.02**

Significant differences are indicated in bold.

**Figure 3:**
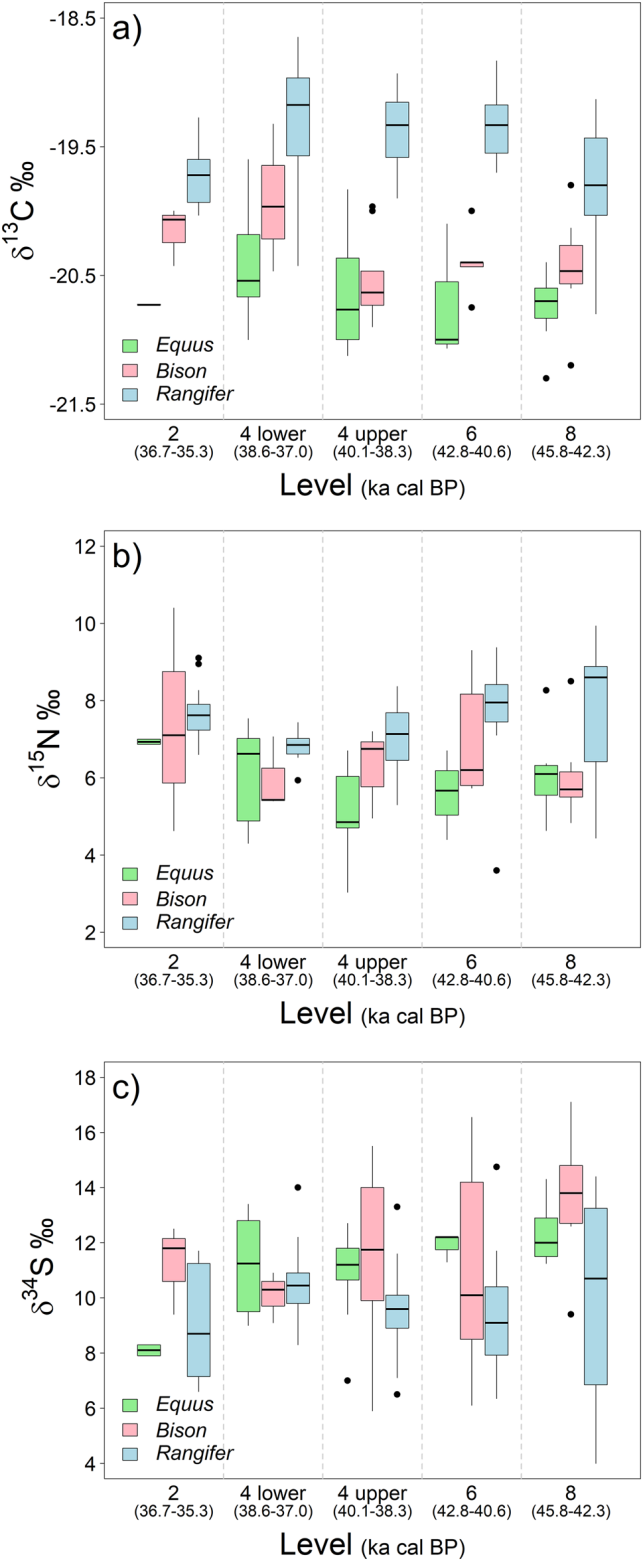
Box plots of all (**a**) carbon, (**b**) nitrogen and (**c**) sulfur isotope data from ungulate bone collagen generated in this study, showing median, quartiles and outliers for each species by level.

The carbon and nitrogen isotope values of all three species, however, demonstrate trends through time, but also variation within each unit at the site between species (see Fig. 3, Fig. S2). To explore these temporal trends, we employed mixed linear models, testing simple and quadratic effects (Table S6–S8). There is a significant quadratic effect for the *δ*^15^N of *Equus* (age^2^: β = 0.09, SE = 0.034, 95% CI [0.02:0.16], Table S7) and almost significant effect for *Rangifer* (age^2^: β = 0.04, SE = 0.021, 95% CI [0.00:0.08], Table S8) in the intermediate levels of the site with the lowest overall *δ*^15^N exhibited in horses in US04 lower, corresponding with the Heinrich 4 event at the site. However, within-species variation in *δ*^15^N is high and inter-species differences are clearer in some levels compared to others (Fig. 3). For example, in the most recent phases of site use (US02 and US04 (upper) there is no significant difference between the three taxonomic groups (Table S5). However, in US04 (lower), US06 and US08 *Rangifer* have significantly higher *δ*^15^N values than *Equus*, and in US08 *Rangifer δ*^15^N are also significantly elevated relative to *Bison* (Table S5).

For *δ*^13^C values, *Rangifer* tend to be elevated compared to other taxa throughout the sequence (Fig. 3), and in pair-wise comparisons are significantly so (compared to *Equus*) in US04 (upper) and compared to both *Equus* and *Bison* in US04 (lower), US06 and US08 (Table S5). While pair-wise comparisons between *Bison* and *Equus* are not significant throughout most of the sequence, *Bison* values tend to be elevated slightly relative to *Equus*. In diachronic comparisons of the data, *Bison* and *Equus* show no age effect with *δ*^13^C values (*Bison*—age: β = 0.04, SE = 0.026, 95%CI [− 0.02:0.09], Table S6; *Equus*—age: β = 0.03, SE = 0.026, 95%CI [− 0.02:0.09], Table S7) whereas carbon isotope values of reindeer are significantly higher in intermediate levels (i.e. US04 upper and lower, and US06, age^2^: β = − 0.03, SE = 0.007, 95% CI [− 0.04:− 0.02], Table S8).

**Figure 4:**
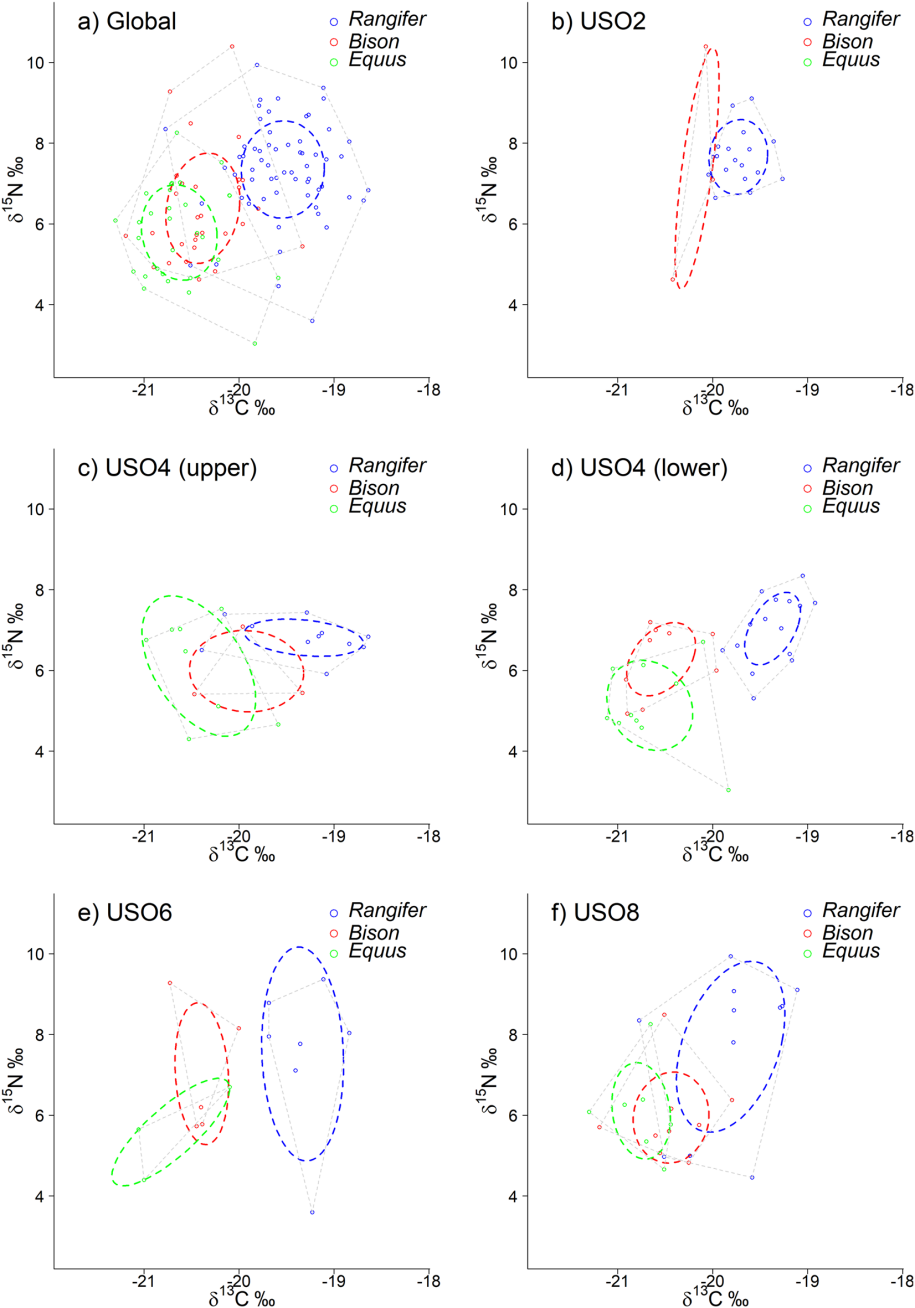
Niche partitioning between *Bison*, *Equus* and *Rangifer* from Les Cottés in the *δ*^13^C–*δ*^15^N isotopic space, for (**a**) all the data and (**b**–**f**) by level. The convex hull area encompassing all the data of a given species (Total Area, TA) is indicated by dashed lines. Ellipses with plain lines correspond to the SEAc (Standard Ellipse Area corrected for small sample size), i.e., the core niche area.

Using isotope niche analysis on the *δ*^13^C–*δ*^15^N isotopic space, we evaluated niche partitioning between species. We looked at the overlap between the total areas (TA) corresponding to the convex hull area containing all the data of each species, and at the overlap between the core areas, defined by the Standard Ellipse Area (SEAc, corrected for small size sample) and containing 40% of the data of each species. Niche partitioning plots are provided in Fig. 4a–f (based on data in Table S9). Overall, across all levels, we observed a complete SEAc core niche partitioning between *Rangifer* and the two other species, while the isotopic core niches of *Equus* and *Bison* moderately overlapped (Fig. 4a). At the different phases of the site, *Rangifer* and *Equus* core niches never overlapped, while TA niches had low overlap (< 30%) in early and late stages (US08 and US04 (upper) respectively (Fig. 4c,f) and no overlap in stages US04 (lower) and US06 (Fig. 4d,e). Niche partitioning mostly occurred with *δ*^13^C values. A similar pattern was observed between *Rangifer* and *Bison* with no overlap for TA and SEAc niches in intermediate stages US04 (lower) and US06 (Fig. 4d,e), and a low (< 30%) to moderate overlap (30–60%) of the *Rangifer* niche by the *Bison* niche during all other site phases (Fig. 4b,c,f). Complete niche partitioning was never observed for *Equus* and *Bison* (Fig. 4b–f) and niches moderately or highly (> 60%) overlapped in most phases, except for TA in phase US06 (Fig. 4e) where the overlap was particularly low.

### Sulfur isotope data

Sulfur values range from 4.0 to 17.1‰, with taxonomic means of 9.7‰ (± 2.4, *Rangifer*), 12.0‰ (± 2.9, *Bison*) and 11.2‰ (± 1.7, *Equus*). Considering all species data across levels, *δ*^34^S values in *Bison* are similar to ẟ^34^S values in *Equus* and both are significantly higher than *δ*^34^S values in *Rangifer* (Table 1). Mixed models reveal that there is a significant decrease through time in *Equus δ*^34^S values (age: β = − 0.32, SE = 0.109, 95% CI [− 0.54:− 0.10], Table S7) and almost significant in *Bison δ*^34^S values (age: β = − 0.37, SE = 0.183, 95% CI [− 0.74:0.00], Table S6), a trend which is not seen in *Rangifer* (age: β = − 0.05, SE = 0.111, 95% CI [− 0.27:0.17], Table S8). However, there are significant differences between the mean variability (i.e., spread of the sulfur data) between taxonomic groups within some levels. Specifically, Levene’s test reveals that variability in *δ*^34^S is significantly different between species in Level US08 (*F* = 3.85, *P* = 0.036), and post-hoc Tukey’s HSD indicates that significant differences in variability in *δ*^34^S are between both *Rangifer* and *Equus* and, to a lesser extent, *Rangifer* and *Bison* but not between *Equus* and *Bison* in this level (Table 2). In the other levels differences in variability between species were not significant (USO4 upper: *F* = 0.71, *P* = 0.507; U04 lower: *F* = 2.35, *P* = 0.113; USO6: *F* = 1.97, *P* = 0.182), or close to significance (USO2: *F* = 2.99, *P* = 0.077), and post-hoc Tukey’s HSD only revealed significant differences in variability in *δ*^34^S between *Rangifer* and *Equus* in Level USO2. Of note, there is no significant correlation between all three isotope systems for any species.

**Table 2:** Post-hoc Tukey’s HSD on the variability *δ*^34^S among species for each levels.

	Estimate	SE	t-value	p-value
USO2				
* Equus—Bison*	− 1.02	0.608	− 1.68	0.234
* Rangifer—Bison*	0.60	0.421	1.42	0.347
*** Rangifer—Equus***	**1.62**	**0.501**	**3.23**	**0.013**
USO4 (upper)				
* Equus—Bison*	0.72	0.6405	1.13	0.505
* Rangifer—Bison*	0.56	0.6307	0.88	0.654
* Rangifer—Equus*	− 0.17	0.4597	− 0.36	0.929
USO4 (lower)				
* Equus—Bison*	− 1.23	0.647	− 1.89	0.157
* Rangifer—Bison*	− 1.19	0.598	− 2.00	0.130
* Rangifer—Equus*	0.03	0.557	0.06	0.998
USO6				
* Equus—Bison*	− 3.03	1.169	− 2.59	0.057
* Rangifer—Bison*	− 1.33	0.937	− 1.42	0.362
* Rangifer—Equus*	1.70	1.105	1.54	0.306
USO8				
* Equus—Bison*	− 0.71	0.836	− 0.85	0.679
* Rangifer—Bison*	1.67	0.713	2.35	0.069
*** Rangifer—Equus***	**2.38**	**0.805**	**2.96**	**0.019**

The estimates correspond to the difference in the mean variability between the compared groups. Significant differences in mean variability between groups are indicated in bold.

## Discussion

Bulk bone collagen carbon and nitrogen isotope ratios from all three taxonomic groups are typical of those from ungulates in C_3_ ecosystems like those found in Late Pleistocene Europe^10,12,23^. However, there are also clear diachronic and taxonomic variations in the dataset which suggest both ecological (behavioural) and environmental variability. Throughout the sequence *Equus* demonstrate values typical of grazers and show no significant variation through time at the site in *δ*^13^C. While differences between *Equus* and *Bison δ*^13^C are not significant throughout most of the sequence, the slight elevation observed in *Bison* relative to *Equus* is to be anticipated between these species even when on the same diet (with methane production leading to a slight tissue ^13^C enrichment in ruminants^58^). The extent to which *Bison* and *Equus* vary from one another, however, does change throughout the sequence (albeit not significantly). As these offsets are not constant, this could imply differences in feeding strategies at different points, with *Bison* exploiting graze and browse to different extends at different times. This would be consistent with both their typical feeding ecology (as intermediate feeders), and also with the environmental variability implied by the presence of temperature/woodland species such as boar, roe deer and red deer in earlier parts of the sequence, and their later absence^2^.

**Figure 5:**
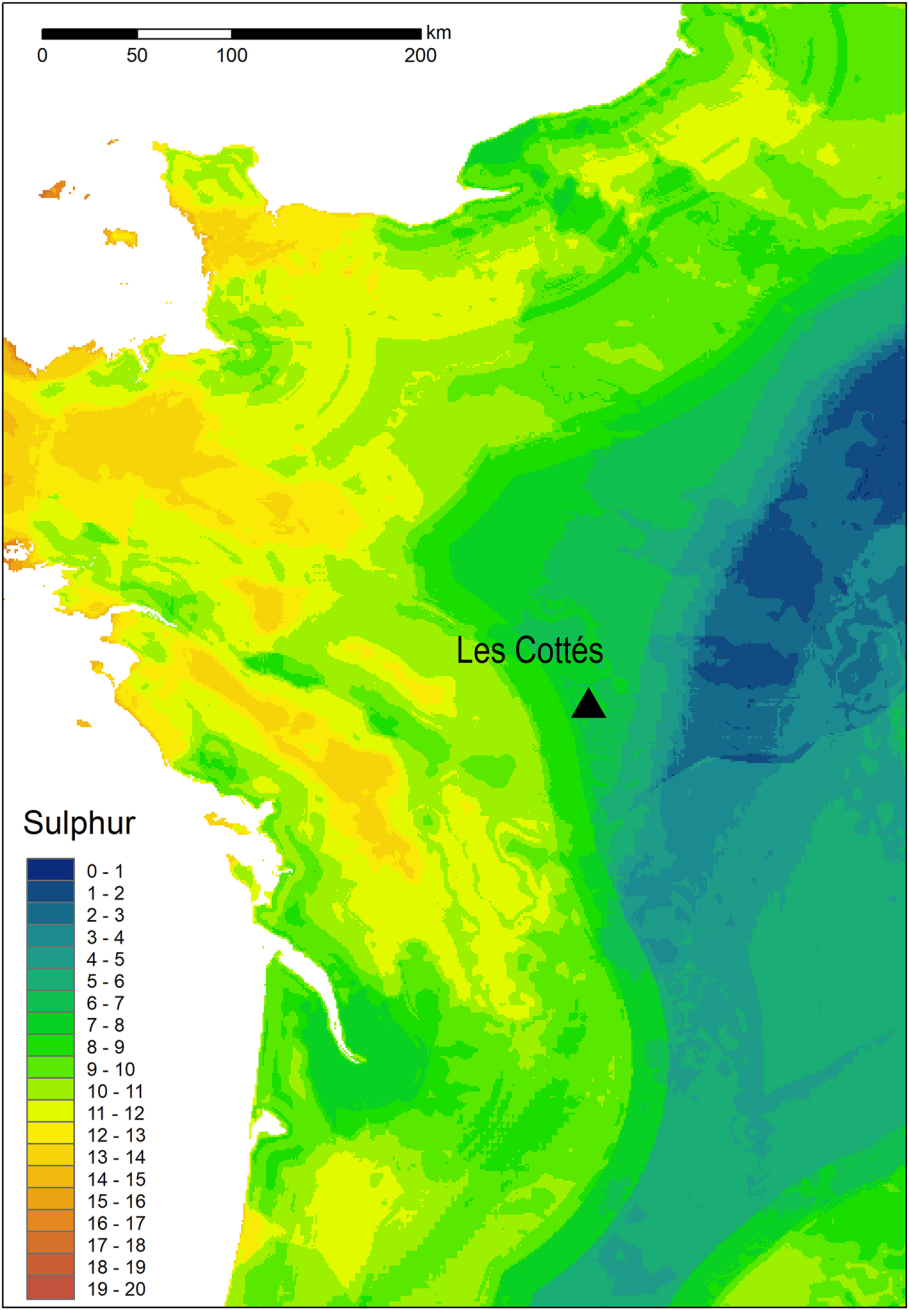
Sulfur isoscape of France. Map created in ArcGIS 10.5 using isoscape data from Bataille et al.^72^.

Compared to the other herbivore species, *Rangifer* bone collagen is consistently and significantly enriched in ^13^C relative to the other species, reflecting the consumption of lichen in this species^43^. While *Equus* and *Bison* do not display notable linear or quadratic effects in mixed models through time, in *Rangifer* there is a pronounced increase in *δ*^13^C values in the intermediate levels of the site, correlating with some of the coldest phases. It is during this time that all species display the lowest *δ*^15^N values. These lower *δ*^15^N values likely reflect broader climatic changes throughout the sequence and indicate that the climate may have been less ameliorate during the formation of US04, which corresponds to the Heinrich 4 event. The *δ*^13^C data suggest that, throughout the sequence, the proportional contribution of lichen in the diet of *Rangifer* was varying. The reasons for this could be due to: (1) the increased (local) availability of lichen and growth of lichen mats during this period, due to cooler, more arid conditions that lichen favour^59^; (2) changes in the extent to which *Rangifer* were exploiting lichens (i.e. to reduce competition between species) and/or (3) a change or increase in *Rangifer* range-size to include regions with a greater abundance of lichens. Given that sulfur isotope ratios are not significantly different between species in any Level aside from US08, a change in movement at this time is perhaps not the most parsimonious interpretation. Furthermore, it can also be noted that in US04 (lower and upper), the range of *δ*^34^S isotope values for *Rangifer* is consistent with local environmental values for the immediate region during the Holocene (Fig. 5) and is more similar to (although slightly lower than) other fauna at the site than it is in other phases. The increased exploitation of lichens remains the most plausible explanation, either through their increased availability and/or due to competition from other ungulates during that period. This provides new direct evidence of behavioural plasticity in *Rangifer* through time, concurrent with climatic changes.

While sulfur values in the three taxonomic groups are most similar in level US04 (upper), there is variation both within and between species observed in other levels of the site, which likely reflect differences in both faunal spatial ecology and in climate. Both *Equus* and *Bison* demonstrate a significant decrease in *δ*^34^S values through time, which may be linked to climatic decline. While the mechanisms of sulfur isotope environmental variability are poorly understood, lower soil values can be found with increasing distance from the coast^60^, but have also been related to lower temperatures and the presence of permafrost, both of which may serve to influence the rate of mineralization and volatilization of sulfur in soils^25,34^. This trend of decreasing *δ*^34^S values, however, is not seen in *Rangifer* although *Rangifer* do have overall lower *δ*^34^S values throughout the sequence, indicating their total range was likely larger and/or includes inland areas to the west and north of the site, which currently exhibit lower environment *δ*^34^S values, reflecting differences in their overall range use^9^. However, given the lack of a contemporary (Pleistocene) sulfur isoscape, and the variability anticipated due to climatic change, environmental pollution and other factors, this should only be considered tentative. Interestingly, the greatest variability in reindeer *δ*^34^S is exhibited in the earliest phases of the site (US08). This may indicate that reindeer spatial ecology was more variable and/or range was more extensive in these earlier phases. This could be related to the period represented in the depositional events of US08 (i.e., be a temporal effect) or could be related to the presence of other deer species at the site during the earliest phases. After US06 (~ 43 k BP) climate is getting colder, lichens become more abundant, and other deer species, such as roe deer and red deer are not found in the record at Les Cottés. This could suggest that in the earliest phases of the site competition for resources was met by a mixture of niche separation and more extensive mobility of *Rangifer*. While there is currently no archaeological evidence to suggest hunting strategies varied substantially through time at Les Cottés, the possibility that hominin hunting range narrowed or broadened to encompass isotopically more homogeneous or varied local areas (i.e. multiple catchments) at different points may also account for some of the diachronic variation observed in the *δ*^34^S of ungulates^8^. Further research, focusing on hominin landscape use and the isotope ecology of diverse deer species and other ungulates at Les Cottés is necessitated. While strontium isotope approaches would be the optimal method for assessing changes in movement behaviours, the presence of only few teeth at the site (reflecting its use as a secondary processing site) limits the usefulness of this approach. The results of this study also underpin the importance of site-specific studies in exploring diachronic change in faunal dietary palaeoecology. Furthermore, the presence of oppositional trends through time at Les Cottés for some species (e.g., ^13^C-enrichment increasing for Rangifer in US04 but decreasing for other species), demonstrates the importance of sampling multiple species when utilising archaeofaunal isotopic variability as a proxy for past environmental conditions.

## Conclusions

The isotope chemistry of bone collagen of *Rangifer*, *Bison* and *Equus* at the Late Pleistocene site of Les Cottés, France, is an archive of changing climatic conditions but also of niche partitioning (and plasticity), over almost a 10,000-year period. While *δ*^15^N values are highly variable, both within species and the same levels, temporal trends broadly correspond with other climatic indicators at the site, with the lowest values corresponding to the Heinrich 4 cooling event in the lower parts of US04. The low *δ*^15^N values in the bone collagen of ungulates during this cold phase agree with previous studies, and likely reflect changes in the composition of soil nitrogen (e.g. due to changes in ammonia volatilisation, age of soil) or plants (e.g. due to nitrogen competition, temperature/aridity effects)^31^. *Bison δ*^13^C values were elevated relative to *Equus*, albeit not significantly so, consistent with ruminant gut physiology and with mixed feeding. The extent to which *Bison* engaged in grazing and browsing behaviours is likely to have varied, both due to vegetation changes and/or presence of other intermediate feeders, such as red deer, as well as reindeer. In contrast, reindeer *δ*^13^C data are more distinct from the other species and display a constant and statistically significant enrichment in ^13^C consistent with lichen consumption. However, the extent to which reindeer are exploiting lichens varies, peaking in US04, likely due to the increase in lichen biomass during this cooler, more arid phase of the site – conditions that favour lichen growth^59^. Due to variation in herbivore isotope values between species and through time at this single site, it is recommended that future studies target multiple species when employing isotope analyses as part of palaeoenvironmental reconstruction, to enable ‘baseline’ and behavioural changes to be better discerned. The range and variability of these data, at a single site, over an approximately ~ 10,000-year period also emphasise the importance of sampling faunal bone from contexts as closely chronologically related to human remains as possible when establishing a faunal baseline in human palaeodietary studies. Given the considerable temporal variability observed here and that seen in larger-scale, multi-site datasets^23,36^, comparison with contemporary (dated) material from other regional sites may even be preferrable to asynchronous material from the same site in such studies.

The *δ*^34^S data generated from bone collagen samples in this study demonstrate the potential of these data to explore diachronic differences in relative range use through time. However, the interactions between environmental change and sulfur isotope environmental variability requires further investigation. Data generated from reindeer in US08 indicate a far greater range of *δ*^34^S values that in other levels for reindeer, indicating not only distinct ranging behaviours compared to the other ungulates in this study but also a greater variability within reindeer. The latter may indicate behaviours were highly variable over a relatively limited time period, again evidencing the behavioural plasticity of this species. The ability of reindeer to migrate larger distances than many other species and to consume lichens likely conferred considerable adaptive advantage during the profound climatic oscillations of the Late Pleistocene period in north-west Europe. The relationship between the movement behaviours and dietary palaeoecology of this species during the Late Pleistocene, and the relationship between plasticity in these behaviours and prevailing climatic conditions, warrants further investigation and may have implications for our understanding of contemporary *Rangifer* communities and their conservation.

## Methods

### Sample selection

Bone samples from selected reindeer (*n* = 68), bovids (*Bison* sp.; *n* = 34) and horses (*Equus* sp.; *n* = 30) from across the five archaeological units US02, US04 upper and lower, US06 and US08. Sampling was limited to bones that could be identified morphologically (conducted by two of the authors, W.R. and S.R.), which did restrict the representation of taxa in certain levels compared to others (see Table 3), although *Rangifer* is well represented throughout. Samples were extracted from larger bones with a clean saw and external surfaces subsequently removed using air abrasion/a tungsten carbide burr. Following initial on-site sampling, samples were prepared and analysed at the Department of Archaeology, University of Aberdeen, and the Max Planck Institute for Evolutionary Anthropology, Leipzig (see SI Table S1).

**Table 3:** Chronological sequence and radiometric dates from Les Cottés^4,48^, and detailing the number of bones sampled from each unit by taxa.

Archaeological Unit—cultural unit	Weighted mean OSL age (ka)	14C age range (68% CI) (ka cal BP)	Taxa by unit—(*NR*) and number sampled (*n*)							
			*Rangifer*		*Bison*				*Equus*	
US02—Upper Early Aurignacian										
	37.2 ± 1.5	36.7–35.3	(*426*)	18		(*7*)	4	(*5*)		2
US04 upper—Early Aurignacian										
	40.5 ± 2.1	38.6–37.0	(*463*)	15		(*20*)	3	(*53*)		8
US04 lower—Protoaurignacian										
	40.3 ± 2.0	40.1–38.3	(*436*)	16		(*43*)	11	(*137*)		10
US06—Châtelperronian										
	43.1 ± 2.2	42.8–40.6	(*95*)	8		(*38*)	5	(*23*)		3
US08—Mousterian										
	51.3 ± 3.0	45.8–42.3	(*127*)	11		(*105*)	11	(*32*)		7
Total				68			34			30

Radiocarbon age ranges exclude those identified in Talamo et al.^4^ as outliers, after Jacobs et al.^48^. Number of Remains (NR) is the number of specimens identifiable to species/taxonomic group is after Rendu et al.^2^ and is noted in italics.

### Laboratory protocols and analysis

All bone samples were prepared following the collagen extraction method of Longin^61^, with modifications based on the recommendations of Collins and Galley^62^, with the addition of an ultrafiltration step^63^. Samples were demineralised in 0.5 M hydrochloric acid at 6–8 °C for 3–10 days with acid changed at regular intervals. They were then rinsed to neutrality with de-ionized water and gelatinized in a weak acidic (pH 3) HCl solution at 70 °C for 48 h. The liquid fraction containing the gelatinized protein was isolated through filtration using 5-8 μm Ezee® mesh filters (Elkay Laboratory Products), and then purified using ultrafilters (> 30,000 kD; Merck Millipore Amicon®). The remaining solution was then frozen and lyophilised. Extraction was successful for 128 of the 132 bones targeted, and the vast majority of samples had collagen yield > 1% and in some cases as high as 4.1%.

Stable carbon and nitrogen isotope measurements on collagen were performed in duplicate in most instances (0.45–0.55 mg of collagen per sample) on a Delta XP mass spectrometer coupled to a Flash EA 2112 elemental analyser in the Department of Human Evolution, Max Planck Institute for Evolutionary Anthropology, Leipzig, Germany. The *δ*^13^C values and *δ*^15^N values are reported relative to the V-PDB standard and AIR standards respectively. Analytical error for the *δ*^13^C and *δ*^15^N measurements was calculated from repeat measurements of internal and international standards and was determined to be ± 0.1‰ (1σ) or better.

For samples with sufficient remaining collagen (n = 126), an additional aliquot of collagen was analysed for stable sulfur (*δ*^34^S) isotopes at the Scottish Universities Research Centre (SUERC). This was conducted on a Delta V Advantage continuous-flow isotope ratio mass spectrometer coupled via a ConfloIV to an IsoLink elemental analyzer (Thermo Scientific, Bremen), enabling the simultaneous measurement of carbon, nitrogen and sulfur stable isotope ratios^64^. At SUERC additional *δ*^13^C and *δ*^15^N measurements, as well as *δ*^34^S measurements, were therefore generated, with an analytical precision of ± 0.3‰ (1 SD) or better for each isotope.

Data generated in both laboratories were considered to be acceptable as all C:N ratios are within the modern range of 3.1 and 3.5^54,56,65^ and the *δ*-values of these samples fall within the main group of the samples. However, a small number of samples presented elemental data with %C < 30% and %N < 11%. If a sample and/or its duplicate from a given laboratory presented low C and N contents not meeting these parameters^54^, all measurements (*δ*^13^C, *δ*^15^N, *δ*^34^S) from that laboratory made on this sample were discarded from further discussion/analysis.

### Statistical analysis

We tested for temporal trends in *δ*^13^C, *δ*^15^N and *δ*^34^S values for each species, using mixed linear models, which was done in order to account for the fact that we had duplicate, triplicate and sometimes quadruplicate analyses for each individual, and from two different laboratories. The random variable was defined as the laboratory ID nested in the individual ID. The age variable was defined as the median value of each level’s age range assessed from radiocarbon dating by Talamo et al.^4^. We tested for linear and quadratic effects of the age, and we selected the model with the lowest AIC (Akaike’s Information Criterion) as the best model. We also tested the differences in *δ*^13^C, *δ*^15^N and *δ*^34^S among species and among levels with mixed models, using the same random effects as for the temporal trend models. For each isotope we ran two models testing: 1) the effect of the species, 2) the interaction between the species and the levels. Pair-wise comparisons were then conducted on the results to compare species and species by levels two by two. Finally, we compared the variability in *δ*^34^S values for each species in each level, using a Levene test followed by a post-hoc Tukey’s HSD (honestly significant difference) test.

All data analyses were implemented in R software (v4.1.2)^66^ using the “lme4”^67^ and “emmeans”^68^ packages for mixed linear models and pair-wise comparison respectively. For all the mixed models, we provided parameter estimates (β) with standard error (SE) and the 95% confidence interval (95%CI). All the models met the assumptions of normality of the residuals and homogeneity of variance.

### Niche analysis

The ecological feeding niche of a species can be inferred using *δ*^13^C and *δ*^15^N isotopes (sometimes referred to as isotopic niche), and be represented on a bivariate plot of *δ*^13^C and *δ*^15^N by the area occupied by the samples of the species^69^. When comparing several species, the degree of overlap between the areas determined for each species indicates if these species occupy similar or different feeding niches^70,71^. We used the “SIBER” R package^71^ to assess the isotopic niche of *Rangifer*, *Equus* and *Bison* at each level when at least 3 samples for a given species were available, and calculated the overlaps between niches. We estimated overlap from two metrics: the convex hull total area^70^ that encompasses all the samples for a given species, and the standard ellipse area, corrected for small sample size (SEAc^71^) corresponding to the core area, i.e., the area that encompasses 40% of the data. We followed Schwartz-Narbonne et al.^23^ to classify the degree of overlap between isotopic niche: low overlap (< 30%), moderate overlap (30–60%), high overlap (> 60%).

### Supplementary Information

Supplementary Information 1.

Supplementary Table S1.

## Data Availability

Isotope data described in the manuscript, the results of all statistical analyses and additional associated figures are provided in the Supplementary Information files.
