# Supplementary material for: Multi-isotope analysis of bone collagen of Late Pleistocene ungulates reveals niche partitioning and behavioural plasticity of reindeer during MIS 3
Source: Sci Rep. 2023 Sep 21;13:15722. doi: 10.1038/s41598-023-42199-7 (PMC10514192; doi:10.1038/s41598-023-42199-7)

Supplementary Information: Multi-isotope analysis of bone collagen from Les Cottés, France, reveals niche partitioning and behavioural plasticity in Late Pleistocene ungulates

Kate Britton[1,2]*, Elodie-Laure Jimenez[1,3+], Mael Le Corre[1+], Sylvain Renou[4,5], William Rendu[6,7], Michael P. Richards[2,8], Jean-Jacques Hublin[2,9] and Marie Soressi[10]

[1]Department of Archaeology, University of Aberdeen, Aberdeen, AB24 3UF, United Kingdom

[2]Department of Human Evolution, Max Planck Institute for Evolutionary Anthropology, 04103, Leipzig, Germany

[3]Royal Belgian Institute of Natural Sciences, 29 Vautier Street, 1000 Brussels, Belgium

[4]Hadès Archéologie, Agence Atlantique, 33100 Bordeaux, France

[5]PACEA, UMR 5199, CNRS, Université de Bordeaux, Ministère de la Culture et de la Communication (MCC), F-33400 Pessac, France

[6]ZooSCAn (IRL 2013) CNRS - IAET SB RAS, Novosibirsk, Russia,

[7]Institut Français des études sur l'Asie Centrale (IFEAC) - UAR 3140 (CNRS-MEAE), Bichkek Kyrgyzstan

[8]Department of Archaeology, Simon Fraser University, 8888 University Drive, Burnaby, BC, Canada V5A 1S6

[9]Collège de France, 11, place Marcelin Berthelot, 74005 Paris

[10]Faculty of Archaeology, Leiden University, 2333CC Leiden, Netherlands

* k.britton@abdn.ac.uk

[+]these authors contributed equally to this work

Table S1 (see Excel File 'SI_Table S1'): $\delta^{13}C$, $\delta^{15}N$ and $\delta^{34}S$ data of *Bison*, *Equus* and *Rangifer* bone collagen samples from Les Cottés archaeological site.

This excel file includes all repeat $\delta^{13}C$, $\delta^{15}N$ and $\delta^{34}S$ measurements of bone collagen samples from Les Cottés, measured at both the Max Planck Institute for Evolutionary Anthropology, Leipzig, (MPI) and the Scottish Universities Environmental Research Centre (SUERC). Includes %C, %N, %S, C:N, C:S and N:S, along with calculated means from duplicate, triplicate or quadruplicate measurements.

Table S2: Difference in $\delta^{13}C$ between species (*Bison*, *Equus* and *Rangifer*) and between species by levels at Les Cottés, estimated with mixed linear models. *Bison* is the reference category for species and US02 for the site level. Significant effects (95% CI excluding 0) are indicated in bold.

Variables	Estimate	SE	t-value	95% CI	
				Lower	Upper
Model : $\delta^{13}C$ ~ Species					
(Intercept)	-20.40	0.076	-268.39	-20.55	-20.25
Equus	**-0.25**	**0.106**	**-2.35**	**-0.46**	**-0.04**
Rangifer	**0.83**	**0.092**	**9.04**	**0.65**	**1.01**
Model : $\delta^{13}C$ ~ Species * Levels					
(Intercept)	-20.19	0.211	-95.79	-20.60	-19.77
Equus	-0.56	0.327	-1.72	-1.21	0.08
Rangifer	0.43	0.227	1.88	-0.02	0.87
US04 (upper)	0.31	0.293	1.07	-0.27	0.89
US04 (lower)	-0.39	0.243	-1.59	-0.87	0.09
US06	-0.21	0.267	-0.79	-0.74	0.32
US08	-0.29	0.242	-1.21	-0.77	0.18
Equus:US04 (upper)	-0.01	0.405	-0.04	-0.81	0.79
Rangifer:US04 (upper)	0.08	0.324	0.24	-0.56	0.72
Equus:US04 (lower)	0.46	0.366	1.25	-0.27	1.18
***Rangifer*:US04 (lower)**	**0.75**	**0.273**	**2.74**	**0.21**	**1.29**
Equus:US06	0.21	0.426	0.49	-0.63	1.05
***Rangifer*:US06**	**0.61**	**0.310**	**1.97**	**0.00**	**1.23**
Equus:US08	0.27	0.373	0.72	-0.47	1.01
Rangifer:US08	0.22	0.277	0.79	-0.33	0.77

Table S3: Difference in $\delta^{15}N$ between species (*Bison*, *Equus* and *Rangifer*) and between species by levels at Les Cottés, estimated with mixed linear models. *Bison* is the reference category for species and US02 for the site level. Significant effects (95% CI excluding 0) are indicated in bold.

Variables	Estimate	SE	t-value	95% CI	
				Lower	Upper
Model : $\delta^{15}N$ ~ Species					
(Intercept)	6.40	0.225	28.50	5.96	6.85
Equus	-0.61	0.315	-1.94	-1.23	0.01
Rangifer	**0.97**	**0.272**	**3.56**	**0.43**	**1.50**
Model : $\delta^{15}N$ ~ Species * Levels					
(Intercept)	7.38	0.651	11.34	6.10	8.67
Equus	-0.44	1.027	-0.43	-2.46	1.59
Rangifer	0.29	0.703	0.41	-1.10	1.67
US04 (upper)	-1.34	0.919	-1.46	-3.16	0.47
US04 (lower)	-1.09	0.752	-1.45	-2.58	0.39
US06	-0.35	0.824	-0.42	-1.97	1.28
US08	-1.43	0.751	-1.90	-2.91	0.06
Equus:US04 (upper)	0.52	1.277	0.41	-2.00	3.04
Rangifer:US04 (upper)	0.48	1.015	0.48	-1.52	2.49
Equus:US04 (lower)	-0.72	1.149	-0.63	-2.99	1.55
Rangifer:US04 (lower)	0.50	0.848	0.59	-1.18	2.17
Equus:US06	-1.02	1.318	-0.77	-3.62	1.58
Rangifer:US06	0.23	0.964	0.24	-1.68	2.13
Equus:US08	0.59	1.172	0.51	-1.72	2.91
Rangifer:US08	1.48	0.865	1.71	-0.23	3.19

Table S4: Difference in δ^{34}S between species (*Bison*, *Equus* and *Rangifer*) and between species by levels at Les Cottés, estimated with mixed linear models. *Bison* is the reference category for species and US02 for the site level. Significant effects (95% CI excluding 0) are indicated in bold.

Variables	Estimate	SE	t-value	95% CI Lower	95% CI Upper
Model : δ^{34}S ~ Species					
(Intercept)	11.99	0.451	26.60	11.10	12.88
Equus	-0.81	0.632	-1.29	-2.06	0.44
Rangifer	**-2.29**	**0.550**	**-4.16**	**-3.38**	**-1.20**
Model : δ^{34}S ~ Species * Levels					
(Intercept)	11.24	1.281	8.77	8.70	13.77
Equus	-3.14	2.025	-1.55	-7.14	0.87
Rangifer	-2.06	1.403	-1.47	-4.83	0.71
US04 (upper)	-1.14	1.812	-0.63	-4.72	2.45
US04 (lower)	0.35	1.502	0.23	-2.62	3.32
US06	-0.14	1.621	-0.09	-3.34	3.06
US08	2.48	1.479	1.68	-0.44	5.40
Equus:US04 (upper)	4.22	2.521	1.68	-0.76	9.21
Rangifer:US04 (upper)	2.57	2.039	1.26	-1.46	6.60
Equus:US04 (lower)	2.42	2.282	1.06	-2.09	6.93
Rangifer:US04 (lower)	0.05	1.707	0.03	-3.32	3.43
Equus:US06	3.94	2.594	1.52	-1.19	9.07
Rangifer:US06	0.51	1.912	0.27	-3.27	4.29
Equus:US08	1.75	2.338	0.75	-2.88	6.37
Rangifer:US08	-1.75	1.722	-1.02	-5.15	1.66

Table S5: Pairwise comparison of the estimates from the mixed linear models testing the differences in δ^{13}C, δ^{15}N and δ^{34}S between species considering all levels together (All Levels model : isotope value ~ species) and between species by level (isotope value ~ species * levels). Significant differences are indicated in bold.

		δ^{13}C					δ^{15}N					δ^{34}S				
Units	Contrast	estimate	SE	df	t-ratio	p-value	estimate	SE	df	t-ratio	p-value	estimate	SE	df	t-ratio	p-value
All Levels	*Bison - Equus*	0.25	0.108	125	2.32	0.06	0.61	0.319	124	1.91	0.14	0.81	0.640	117	1.27	0.42
	Bison - Rangifer	**-0.83**	**0.093**	**125**	**-8.92**	**<0.001**	**-0.97**	**0.275**	**124**	**-3.51**	**<0.01**	**2.29**	**0.558**	**117**	**4.11**	**<0.001**
	Equus - Rangifer	**-1.08**	**0.091**	**123**	**-11.80**	**<0.001**	**-1.58**	**0.272**	**124**	**-5.79**	**<0.001**	**1.48**	**0.551**	**117**	**2.68**	**0.02**
US02	*Bison - Equus*	0.56	0.349	139	1.61	0.25	0.44	1.097	138	0.40	0.92	3.14	2.173	131	1.44	0.32
	Bison - Rangifer	-0.43	0.242	145	-1.76	0.19	-0.29	0.751	139	-0.38	0.92	2.06	1.506	131	1.37	0.36
	Equus - Rangifer	**-0.99**	**0.282**	**134**	**-3.50**	**<0.001**	-0.72	0.894	138	-0.81	0.70	-1.08	1.792	131	-0.60	0.82
US04 (upper)	*Bison - Equus*	0.58	0.256	134	2.25	0.07	-0.08	0.812	137	-0.10	0.99	-1.09	1.612	131	-0.67	0.78
	Bison - Rangifer	-0.51	0.247	135	-2.05	0.11	-0.77	0.782	138	-0.99	0.59	-0.51	1.587	131	-0.32	0.94
	Equus - Rangifer	**-1.08**	**0.177**	**137**	**-6.12**	**<0.001**	-0.69	0.558	138	-1.23	0.44	0.58	1.157	131	0.50	0.87
US04 (lower)	*Bison - Equus*	0.11	0.176	140	0.60	0.82	1.16	0.552	138	2.10	0.09	0.72	1.130	131	0.63	0.80
	Bison - Rangifer	**-1.18**	**0.162**	**142**	**-7.24**	**<0.001**	-0.78	0.507	139	-1.55	0.27	2.01	1.043	131	1.92	0.14
	Equus - Rangifer	**-1.28**	**0.155**	**134**	**-8.29**	**<0.001**	**-1.94**	**0.490**	**137**	**-3.97**	**<0.001**	1.29	0.972	131	1.33	0.38
US06	*Bison - Equus*	0.35	0.290	156	1.21	0.45	1.45	0.882	141	1.65	0.23	-0.80	1.740	131	-0.46	0.89
	Bison - Rangifer	**-1.04**	**0.226**	**142**	**-4.60**	**<0.001**	-0.51	0.704	139	-0.73	0.75	1.55	1.394	131	1.11	0.51
	Equus - Rangifer	**-1.39**	**0.272**	**153**	**-5.11**	**<0.001**	**-1.97**	**0.832**	**140**	**-2.36**	**0.05**	2.35	1.644	131	1.43	0.33
US08	*Bison - Equus*	0.29	0.192	137	1.53	0.28	-0.16	0.605	138	-0.26	0.96	1.39	1.255	131	1.11	0.51
	Bison - Rangifer	**-0.64**	**0.170**	**134**	**-3.79**	**<0.001**	**-1.76**	**0.539**	**137**	**-3.27**	**<0.001**	**3.81**	**1.071**	**131**	**3.55**	**0.00**
	Equus - Rangifer	**-0.94**	**0.184**	**137**	**-5.09**	**<0.001**	**-1.61**	**0.580**	**138**	**-2.77**	**0.02**	2.42	1.209	131	2.00	0.12

Table S6: Changes over time in $\delta^{13}C$, $\delta^{15}N$ and $\delta^{34}S$ for *Bison*. Linear and quadratic effects of the median age of the level were estimated using mixed linear models. Best models are indicated in bold with their AICc, as well as significant effects.

model	AICc	variable	estimate	se	t-value	95% CI	
						lower	upper
**$\delta^{13}C$ ~ age**	**-1.3**	intercept	-18.93	1.059	-17.88	-21.07	-16.78
		age	0.04	0.026	1.39	-0.02	0.09
$\delta^{13}C$ ~ age + age²	-0.1	intercept	0.03	18.079	0.00	-36.47	36.65
		age	0.98	0.894	1.09	-0.83	2.79
		age²	0.01	0.011	1.05	-0.01	0.03
**$\delta^{15}N$ ~ age**	**87.6**	intercept	10.04	3.623	2.77	2.70	17.39
		age	0.09	0.089	1.01	-0.09	0.27
$\delta^{15}N$ ~ age + age²	89.9	intercept	17.90	62.002	0.29	-107.81	143.48
		age	0.48	3.068	0.16	-5.74	6.69
		age²	0.00	0.038	0.13	-0.07	0.08
**$\delta^{34}S$ ~ age**	**148.4**	intercept	-3.03	7.477	-0.41	-18.21	12.14
		**age**	**-0.37**	**0.183**	**-2.01**	**-0.74**	**0.00**
$\delta^{34}S$ ~ age + age²	150.2	intercept	125.10	126.335	0.99	-131.26	381.46
		age	5.98	6.255	0.96	-6.71	18.68
		age²	0.08	0.077	1.02	-0.08	0.23

Table S7: Changes over time in $\delta^{13}C$, $\delta^{15}N$ and $\delta^{34}S$ for *Equus*. Linear and quadratic effects of the median age of the level were estimated using mixed linear models. Best models are indicated in bold with their AICc, as well as significant effects.

model	AICc	variable	estimate	se	t-value	95% CI	
						lower	upper
**$\delta^{13}C$ ~ age**	**-27.2**	intercept	-19.38	1.060	-18.28	-21.53	-17.24
		age	0.03	0.026	1.19	-0.02	0.09
$\delta^{13}C$ ~ age + age²	-24.9	intercept	-22.67	20.281	-1.12	-63.74	18.39
		age	-0.13	1.001	-0.13	-2.16	1.90
		age²	0.00	0.012	-0.16	-0.03	0.02
$\delta^{15}N$ ~ age	-6.3	intercept	6.15	3.242	1.90	-0.42	12.71
		age	0.01	0.081	0.11	-0.15	0.17
**$\delta^{15}N$ ~ age + age²**	**-10.3**	intercept	153.08	55.895	2.74	39.92	266.23
		**age**	**7.27**	**2.759**	**2.64**	**1.68**	**12.85**
		**age²**	**0.09**	**0.034**	**2.63**	**0.02**	**0.16**
**$\delta^{34}S$ ~ age**	**113.0**	intercept	-1.53	4.344	-0.35	-10.33	7.28
		**age**	**-0.32**	**0.109**	**-2.93**	**-0.54**	**-0.10**
$\delta^{34}S$ ~ age + age²	114.4	intercept	-93.77	78.678	-1.19	-253.24	65.71
		age	-4.88	3.887	-1.26	-12.76	3.00
		age²	-0.06	0.048	-1.17	-0.15	0.04

Table S8: Changes over time in $\delta^{13}C$, $\delta^{15}N$ and $\delta^{34}S$ for *Rangifer*. Linear and quadratic effects of the median age of the level were estimated using mixed linear models. Best models are indicated in bold with their AICc, as well as significant effects.

model	AICc	variable	estimate	se	t-value	95% CI lower	95% CI upper
$\delta^{13}C$ ~ age	35.6	intercept	-19.35	0.734	-26.36	-20.81	-17.89
		age	0.01	0.019	0.30	-0.03	0.04
**$\delta^{13}C$ ~ age + age²**	**22.1**	intercept	-64.94	10.780	-6.02	-86.40	-43.47
		**age**	**-2.29**	**0.542**	**-4.22**	**-3.37**	**-1.21**
		**age²**	**-0.03**	**0.007**	**-4.24**	**-0.04**	**-0.02**
$\delta^{15}N$ ~ age	158.1	intercept	6.35	2.042	3.11	2.28	10.41
		age	-0.03	0.052	-0.50	-0.13	0.08
**$\delta^{15}N$ ~ age + age²**	**156.5**	intercept	71.31	33.138	2.15	5.35	137.28
		age	3.24	1.666	1.95	-0.07	6.56
		**age²**	**0.04**	**0.021**	**1.96**	**0.00**	**0.08**
**$\delta^{34}S$ ~ age**	**283.4**	intercept	7.84	4.396	1.78	-0.92	16.60
		age	-0.05	0.111	-0.42	-0.27	0.17
$\delta^{34}S$ ~ age + age²	285.6	intercept	-21.08	72.837	-0.29	-166.25	124.14
		age	-1.50	3.656	-0.41	-8.79	5.79
		age²	-0.02	0.046	-0.40	-0.11	0.07

Table S9: Percentage of overlap between the isotopic niches of *Rangifer, Bison and Equus* defined within the $\delta^{13}C$ - $\delta^{15}N$ isotopic space. TA (Total Area) corresponds to the convex hull area encompassing all the data of a given species. SEAc (Standard Ellipse Area corrected for small sample size) refers the core niche area. Low overlap (<30%) is indicated in italic, and high overlap (>60%) in bold.

Unit	Species 1	Species 2	% of TA overlap sp.1 by sp.2	% of TA overlap sp.2 by sp.1	% of SEAc overlap sp.1 by sp.2	% of SEAc overlap sp.2 by sp.1
All Levels	*Rangifer*	*Bison*	48.3	**67.9**	*0*	*0*
	Rangifer	*Equus*	*26.5*	46.7	*0*	*0*
	Bison	*Equus*	51.2	**64.2**	48.6	55.9
US02	*Rangifer*	*Bison*	*1.0*	*1.6*	*2.4*	*0.4*
US04 (upper)	*Rangifer*	*Bison*	*12.3*	*23.3*	47.6	*11.5*
	Rangifer	*Equus*	*12.9*	*8.8*	*0*	*0*
	Bison	*Equus*	53.8	*19.5*	37.3	**76.1**
US04 (lower)	*Rangifer*	*Bison*	*0*	*0*	*0*	*0*
	Rangifer	*Equus*	*0*	*0*	*0*	*0*
	Bison	*Equus*	**64.7**	33.6	37.3	*24.5*
US06	*Rangifer*	*Bison*	*0*	*0*	*0*	*0*
	Rangifer	*Equus*	*0*	*0*	*0*	*0*
	Bison	*Equus*	*6.0*	*11.4*	44.2	33.2
US08	*Rangifer*	*Bison*	30.1	**69.5**	*9.1*	*20.3*
	Rangifer	*Equus*	*5.7*	*22.9*	*0*	*0*
	Bison	*Equus*	41.6	**72.8**	41.9	50.6

Figure S1: Differences in a) $\delta^{13}C$, b) $\delta^{15}N$, c) $\delta^{34}S$) between species (*Bison*, *Equus*, *Rangifer*) at Les Cottés.

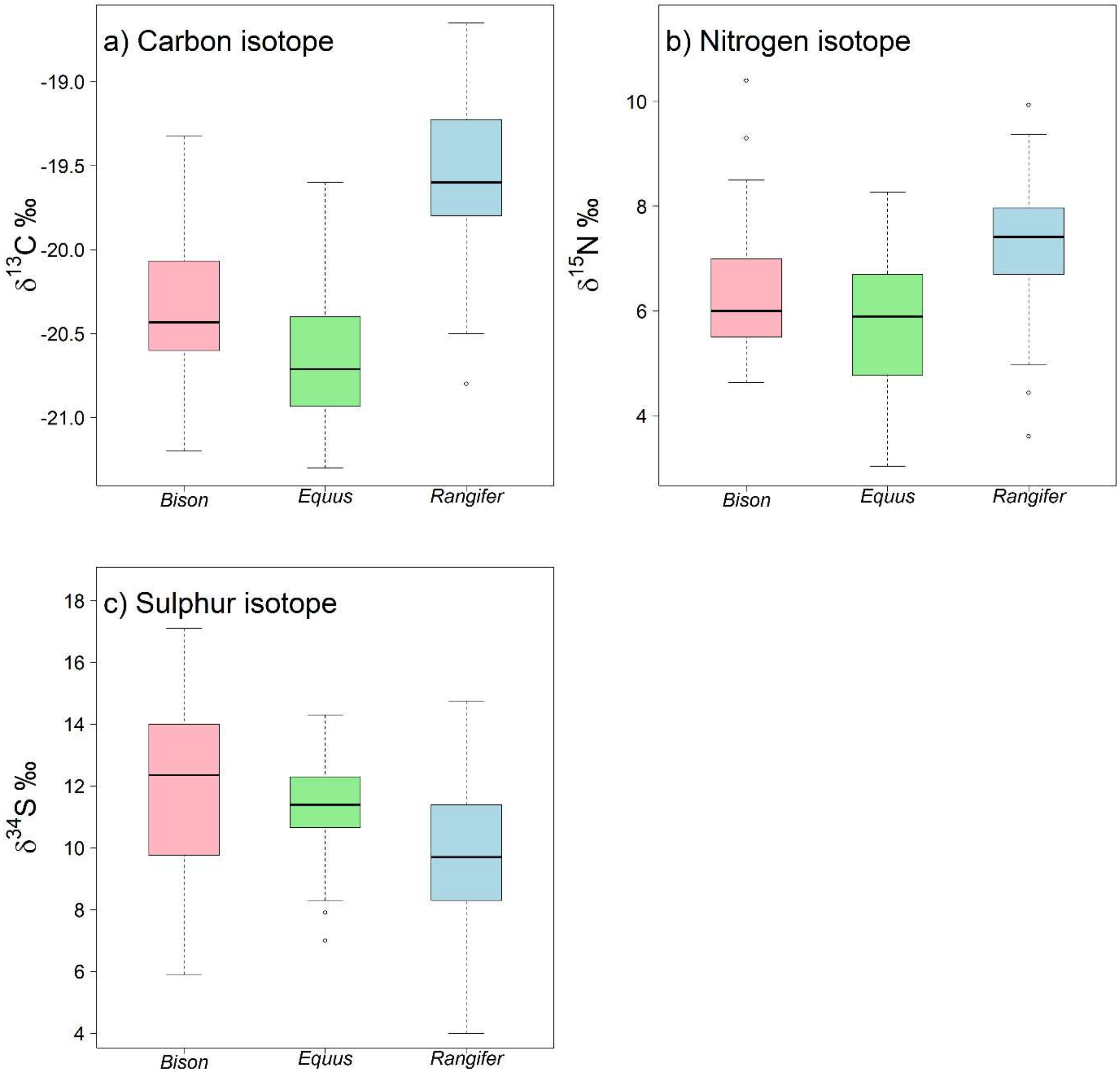

Figure S2: Box plots of a) carbon and c) nitrogen data from ungulate bone collagen generated at the Max Planck Institute, Liepzig (MPI) and b) carbon and d) nitrogen data generated at the Scottish Universities Research Centre (SUERC) from the same bone samples. Box plots show the median isotope values, quartiles and outliers for each species by level.

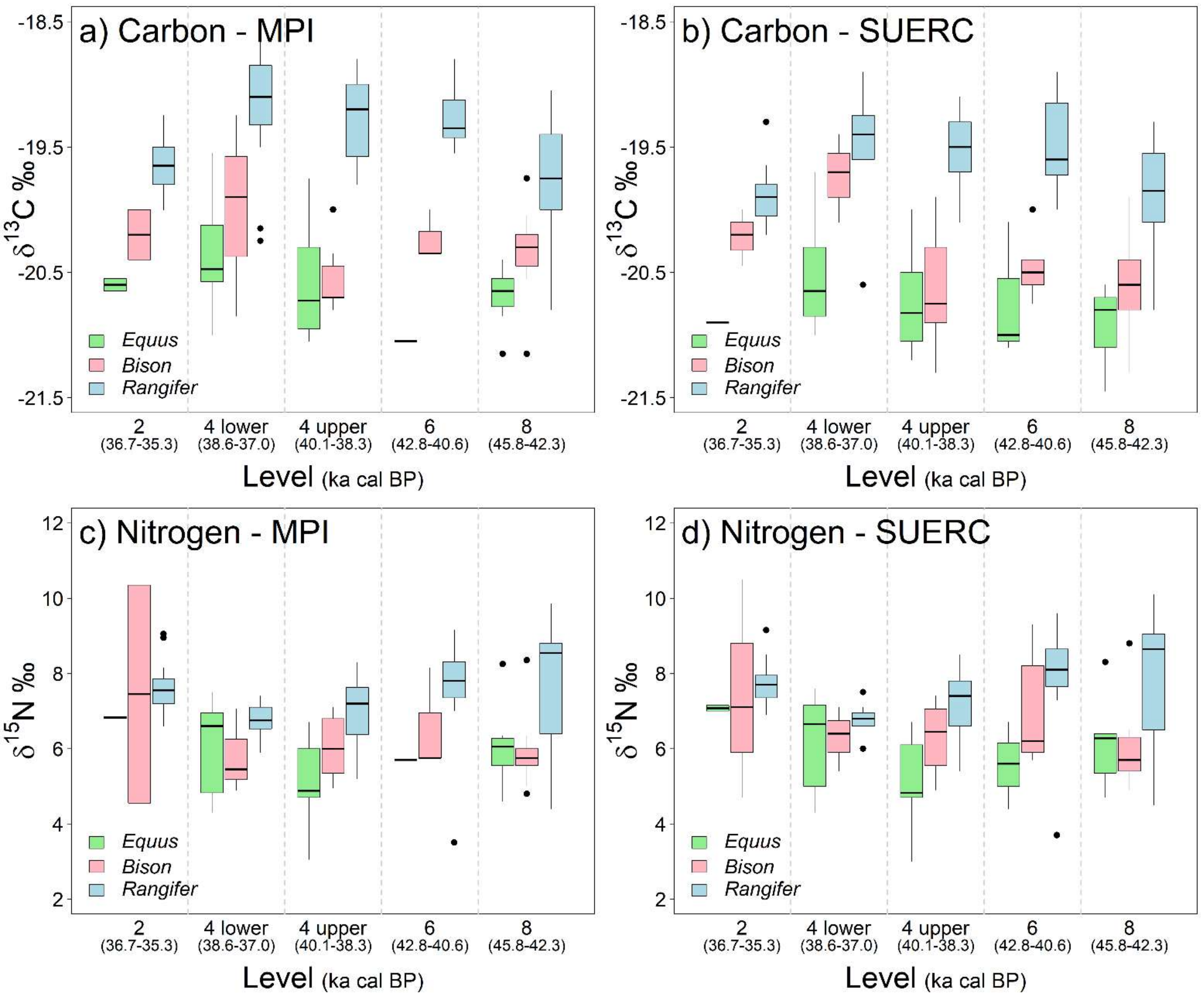

Supplement: Supplementary file 1 — Supplementary Information 1. [file 41598_2023_42199_MOESM1_ESM.pdf]
